# Functional Brain Connectivity During Narrative Processing Relates to Transportation and Story Influence

**DOI:** 10.3389/fnhum.2021.665319

**Published:** 2021-07-05

**Authors:** Anthony G. Vaccaro, Brandon Scott, Sarah I. Gimbel, Jonas T. Kaplan

**Affiliations:** ^1^Department of Psychology, Brain and Creativity Institute, University of Southern California, Los Angeles, CA, United States; ^2^Department of Psychology, Yale University, New Haven, CT, United States

**Keywords:** fMRI, narrative, narrative transportation, naturalistic stimuli, functional connectivity, MVPA

## Abstract

Engaging with narratives involves a complex array of cognitive and affective processes. These processes make stories persuasive in ways that standard arguments are not, though the underlying reasons for this remain unclear. Transportation theory proposes a potential explanation for this: narratives are processed in a way which makes individuals feel immersed in the world of a story, which in turn leads people to resonate emotionally with the events of the story. Recent fMRI studies have shown that the posterior medial cortex (PMC) and anterior insula (AI) play important roles in understanding the meaning of stories and experiencing the feelings they produce. In this study, we aimed to explore the AI’s and PMC’s role in narrative processing by measuring their functional connectivity with the rest of the brain during story listening, and how connectivity changes as a function of narrative transportation and the persuasiveness of the story. We analyzed data from 36 right-handed subjects who listened to two stories, obtained from podcasts, inside the fMRI scanner. After the scan, subjects were asked a series of questions, including a measure of how transported into the story they felt, how likely they would be to donate to causes related to the messages of the stories. We used searchlight multivariate pattern analysis (MVPA) to classify functional connectivity maps using seeds in both the AI and PMC and to compare these maps between participants who differed in transportation and prosocial intention. We found that connectivity to various regions successfully distinguished between high and low ratings on each of these behavioral measures with accuracies over 75%. However, only one pattern of connectivity was consistent across both stories: PMC-inferior frontal gyrus connectivity successfully distinguished high and low ratings of narrative transportation in both stories. All other findings were not consistent across stories. Instead, we found that patterns of connectivity may relate more to the specific content of the story rather than to a universal way in which narratives are processed.

## Introduction

Recent research suggests that messages in narrative form may be more persuasive compared with standard arguments (Deighton et al., 1989; Slater and Rouner, 2002; Bilandzic and Busselle, 2013). The underlying reasons for the persuasiveness of stories remain unclear, but likely depend upon the specific cognitive and affective mechanisms involved in story comprehension. Whereas processing a standard argument requires focusing on a sequence of facts and arguments, processing stories requires attention to characters, their intentions, and sequences of events. Furthermore, stories often evoke affect in ways standard arguments do not. One prominent hypothesis as to why stories are generally more persuasive in their messages is transportation theory (Green and Brock, 2000; Green and Sestir, 2017). Transportation theory posits that the way narratives are processed can make individuals feel as if they immersed in the world of a story (Green et al., 2004; Batat and Wohlfeil, 2009; Van Laer et al., 2014). Being immersed then causes people to react emotionally in a way that is similar to how they react to real-life events. This is thought to make the listener/reader more likely to be persuaded of the story’s message (Escalas, 2004; Green et al., 2004; Banerjee and Greene, 2012; Green and Sestir, 2017; Morris et al., 2019). Narrative transportation can facilitate persuasion where individuals’ beliefs are typically resistant to change, such as in regard to health-related behaviors (Banerjee and Greene, 2012; Green and Clark, 2013; Dillard et al., 2018), intergroup prejudice (Mazzocco et al., 2010; Caputo and Rouner, 2011; Johnson et al., 2013; Guerrero and Igartua, 2017; Moyer-Gusé et al., 2019) and prosocial decision-making (Harjusola-Webb et al., 2012; Johnson, 2012; Steinemann et al., 2017).

Narrative transportation is influenced by individual state and trait differences in affect. For example, when individuals are in an affective state that matches the emotional tone of the story they are about to experience, the reported feeling of transportation increases (Green et al., 2012). On the trait-level, individuals who actively seek out emotion-inducing situations are more likely to feel transported into a narrative and more likely to be persuaded by the narrative (Appel and Richter, 2010). Trait empathy also correlates with the tendency to be transported into narratives: transported readers experience more empathy toward characters they read about (Green and Donahue, 2009; Hall and Bracken, 2011; Weiss, 2015).

Individuals who feel transported into a story identify more with the story’s characters, displaying more of their traits and emotions (Green et al., 2004; Johnson et al., 2014; Christy and Fox, 2016; Igartua and Frutos, 2017). They also report temporary differences in their self-concept which align more with the characters (Sestir and Green, 2010; Breen et al., 2017). Some highly transportable people may be motivated to engage with narrative media in order to temporarily redefine their self-concept as more alike to a narrative world’s character (Hirschman, 1983; Greenwood and Long, 2009; Klimmt et al., 2009; Li et al., 2011; Slater et al., 2014; Christy and Fox, 2016; Kuo et al., 2016). Narratives may also trigger autobiographical memories, which are known to play an important role in defining sense of self (Dunlop et al., 2010; Prebble et al., 2013; Rasmussen et al., 2014; McDonald et al., 2015) and are organized in narrative form (Robinson and Taylor, 1998; Conway et al., 2004; Singer and Blagov, 2004; Fivush, 2011; Prebble et al., 2013). For this reason, processing the structure of narratives in media may be an effective way to prime specific autobiographical memories. This would then affect one’s current sense of self by highlighting specific feelings related to that memory (Baumgartner et al., 1992; Moore and Homer, 2008; McDonald et al., 2015; Hartmann et al., 2016).

During the last few years there has been an increase in fMRI studies focusing on narratives. Many of these studies have highlighted an important role for the default mode network (DMN), and in particular, the posterior medial cortex (PMC) (Fransson and Marrelec, 2008). PMC regions (which include the mesial precuneus, posterior cingulate cortex, and retrosplenial areas) are known to be important for self-related processes and self-concept (Seger et al., 2004; Parvizi et al., 2006; Summerfield et al., 2009; Rameson et al., 2010; Araujo et al., 2015; Feng et al., 2018). Given the PMC’s role in autobiographical self-related processes, it is no surprise that these regions would also be relevant to narrative processing. The DMN, including the PMC, appears to be involved in the process of making sense out of long form event sequences (Lerner et al., 2011; Baldassano et al., 2017, 2018). Inter-subject correlation analyses have found that the specific meanings of narratives can be decoded across subjects by the spontaneous correlation patterns within BOLD activity in the DMN and PMC regions (Hasson et al., 2008; Simony et al., 2016; Nguyen et al., 2019). Furthermore, searchlight multi-voxel pattern analysis can accurately predict shared meanings of stories presented to different subjects in their native languages, and even the higher-level moral values of these stories (Dehghani et al., 2017; Kaplan et al., 2017). Euclidian distance-based classification has shown that BOLD activity in PMC regions can predict people’s specific interpretations of stories as well, demonstrating how closely this area corresponds to the phenomenological experiences of narratives (Yeshurun et al., 2017).

Another region which may be relevant to the experience of narratives is the anterior insula. The anterior insula (AI) is known to integrate homeostatic information from the body and is foundational to the conscious subjective experience of emotions (Craig, 2009; Zaki et al., 2012; Gu et al., 2013). This integrative role has been shown to be operative while listening to narratives. In one such study, effective connectivity was used to show that the AI was integrating audio information from the narrative with heart rate variability information from the posterior insula, as well as displaying more activity in general during emotionally salient parts of a story (Nguyen et al., 2016). The insula’s role in integrating information for emotional experience may also lead its activity to reflect the perceived vividness of stories (Bruneau et al., 2013).

Importantly, AI activity has been found to correlate with differences in experiences between narrative consumers. AI activity correlates with various types of admiration for a narrative’s protagonist (Immordino-Yang et al., 2009), and AI activity has also been used to differentiate cultural influences on how narrative-induced feelings are formed. For instance, while the ventral AI (which modulates the autonomic nervous system) is associated with how admirable Chinese listeners felt the protagonists in a story were, the dorsal AI (which relates to somatosensory and visceral input) associated with how admirable Americans felt the characters were (Immordino-Yang et al., 2014). Furthermore, regardless of cultural background, the strength of the correlation between admiration and the dorsal AI was moderated by how expressive the person was in a previous interview about the narrative (Immordino-Yang et al., 2016). Culturally specific processes of feeling explain why stories written within a certain culture are more likely to influence transportability and be persuasive: the language of stories reflect these distinctions in how emotions are subjectively felt in different cultures (Stolte and Fender, 2007; Larkey and Hecht, 2010; Swanson et al., 2017).

Despite consistent findings implicating the PMC and AI in narrative processing, there has been little research into the functional connectivity of these regions while listening to narratives. The AI (Jabbi et al., 2008; Cauda et al., 2011) and PMC (Fransson and Marrelec, 2008; Zhang and Chiang-shan, 2012; Khalsa et al., 2014) function as nodes within neural networks rather than as isolated regions. By understanding how they function within broader neural networks while listening to stories, we can learn more about what cognitive and affective processes help facilitate the effects previously discussed, such as the feeling of transportation and the increased persuasive influence. Furthermore, functional connectivity analysis lends itself well to the study of complex, naturalistic stimuli such as stories.

Our first set of hypotheses concerned functional connectivity while listening to stories relating to behavioral characteristics of the individual listeners. We predicted that functional connectivity patterns would differ between those who were transported into the stories and those who weren’t. Likewise, we predicted that functional connectivity patterns would differ between subjects who were persuaded to support causes and those who weren’t.

We also hypothesized that AI and PMC connectivity to the broader DMN would be predictive of transportation and of the **story-specific** prosocial intentions (i.e., for a story involving cancer, the intention to donate to cancer research rather than to another cause). Increased connectivity between these two seeds and other DMN regions, such as the medial prefrontal cortex and angular gyrus, may reflect the emotional impact of the story having more influence on self-other boundaries, and thus reflect the persuasive influence of the story. Specifically, we chose to focus on the dorsal AI due to its unique role in modulating the DMN compared to other insular sub-regions (Nomi et al., 2016; Uddin et al., 2017), and its role integrating somatosensory and visceral input with other cognitive and affective functions, which we believe is particularly relevant to these stories (Deen et al., 2010; Uddin et al., 2014). Finally, we hypothesized that AI and PMC connectivity to sensorimotor areas would be additionally predictive of narrative transportation due to the individuals feeling as-if they are situated in the actions and sensations of the story.

We used searchlight-based multi-voxel pattern analysis (MVPA) to compare connectivity maps between groups of subjects. Because signal from many voxels are considered simultaneously, MVPA shows increased sensitivity compared with traditional univariate analysis (Kriegeskorte et al., 2006; Norman et al., 2006; Kriegeskorte and Bandettini, 2007). Our first set of hypotheses would be supported if our MVPA of the functional connectivity maps can accurately classify the behavioral characteristics we are measuring (high vs. low transportation and intention to support causes). The strongest outcome for the rest of our hypotheses would be if these patterns of connectivity were then consistent across both stories. A pattern of connectivity in one story but not the other may suggest a process specific to the story’s content rather than a general mechanism universal for stories.

## Materials and Methods

All procedures were approved by the Institutional Review Board of University of Southern California.

### Participants

Forty-five right-handed, native-English speaking subjects were recruited for this fMRI study from the Los Angeles area. However, after removing three subjects for excessive movement (movements >1.5 mm from one volume to the next), one for falling asleep during the scan, one for an incidental finding in the brain, as well as another four subjects for failing basic comprehension questions about the stories they listened to, 36 subjects remained (18 female, mean age: 22.30 ± 0.83 years) (see Table 1). Subjects had no history of neurological disorder or of psychiatric disorder (as measured by the Neuropsychiatric Inventory Questionnaire).

**TABLE 1:** Participant demographics.

Demographics Table					

	Mean	Standard Deviation	Range		
**Age**	22.3	0.83	18–39		

	**Female**	**Male**			

**Gender**	18	18			

	**Asian**	**Black**	**Filipino**	**Hispanic**	**White**

**Race/Ethnicity**	8	4	3	5	16

	**Christian/Catholic**	**Christian/Protestant**	**Christian/Other**	**Jewish**	**None**

**Religion**	9	6	5	2	14

### Stimuli

Two stories were selected from popular podcasts. These two stories were “The Living Room” from Love + Radio podcast and “The Hitcher” from This American Life. In “The Living Room,” the protagonist describes watching a young couple over time through their open window. Eventually, one member of the couple gets cancer and the protagonist describes watching them decline over time, eventually succumbing to the disease. In “The Hitcher,” two young brothers hitchhike to get home one night. Along the way, the driver brings them to various locations and the brothers are unsure if they are being kidnapped before they eventually decide to escape. Each story was transcribed from the podcast and then read aloud by a professional voice actor. “The Living Room” was read by a female actor while “The Hitcher” was read by a male actor.

### Procedure

Participants were given instructions to listen to the stories with their eyes open and to lie still in the scanner. Each participant started with a resting state scan (not analyzed here), followed by the two stories, counterbalanced in order across the subjects. Each story started with 10 s of silence. The stories were “The Living Room” (11 min, 42 s), originally from Love + Radio podcast), and “The Hitcher” (10 min, 5 s), originally from This American Life). Participants listened to the stories through a set of fMRI-compatible in-ear headphones (model S14, Sensimetrics). The volume was calibrated for each subject to ensure they could hear the stories over the noise of the scanner. The participants’ eyes were monitored by the experimenter in real-time with an eye tracking camera to ensure wakefulness throughout the story scans.

After the scan, subjects answered comprehension questions to assure they were actively listening to the stories. Subjects also answered the questions of the Transportation Scale Short Form (TS-SF) relative to each story (Appel et al., 2015). Each item on this scale is rated from 1 (not at all) to 7 (very much) and averaged for a total score. Finally, subjects answered various questions about how willing they were to contribute to various causes on a scale from 0 to 10. Relevant to the current analyses, there was a question related to the theme of “The Living Room,” and a question related to the theme of “The Hitcher” as follows:

“How willing are you to donate $5 to a cancer research organization?”
“How willing are you sign a petition supporting the education of minors on the dangers of hitchhiking?”

### Imaging Parameters

A 3T Siemens MAGNETON Prisma system with a 32-channel head coil was used for the fMRI portion of the study (located at the Dana and David Dornsife Neuroscience Institute at University of Southern California). T1-weighted scans were obtained at 1 mm × 1 mm × 1 mm resolution using a 3D magnetization-prepared rapid acquisition gradient (MPRAGE) sequence. One-hundred seventy-six slices were obtained with a 10-degree flip angle, 256 × 256 matrix, and a phase encoding direction of right to left. TR was 2,300 ms and TE was 2.26 ms.

Functional images were obtained with a gradient-echo, echo-planar T2^∗^-weighted multiband pulse sequence. Forty-eight slices 3 mm × 3 mm × 3 mm resolution slices were obtained with a 90-degree flip angle, 64 × 64 matrix. TR was 1,000 ms and TE was 35 ms, in interleaved ascending order. For “The Living Room” 740 functional volumes were obtained and for “The Hitcher” 625 functional volumes were obtained. The gradient-echo field map was also obtained, to be used for later field-inhomogeneity correction, with a 90-degree flip angle and 64 × 64 matrix. TR was 1,000 ms, TE1 was 10 ms, and TE2 was 12.45 ms.

### Image Pre-processing

Pre-processing and seed-connectivity analyses were performed using FSL version 5.0.8^1^. The gradient-echo field map was used to correct magnetic field inhomogeneity along with FSL’s FUGUE tool for unwarping EPI’s (anterior-posterior direction, 10% signal loss threshold). Motion-correction was performed with a rigid-body alignment to the middle volume of the scan as a point of reference. Slice-time correction was performed with Fourier-space time series phase shifting. Skull-stripping was performed using FSL’s BET brain extraction tool with the center voxel of the brain manually specified (fractional intensity threshold = 0.4). Spatial smoothing was performed with a 5 mm FWHM kernel. High-pass temporal filtering was applied with a Gaussian weighted least-squares line (sigma = 60 s) and temporal autocorrelation was accounted for with FSL’s pre-whitening algorithm. In order to remove the effects of the onset and offset of the audio stimulus, which could drive significant variability in the signal, we removed 16 volumes from the beginning (10 s of silence + 6 for hemodynamic delay) and four volumes from the end of each dataset.

Functional images were registered to their respective T1 images using FSL FLIRT’s boundary-based registration algorithm. Finally, the images were registered to a common MNI 152 space with 12 degree of freedom affine transformation and FNIRT registration (warp resolution = 10 mm).

### Data Analysis

#### Functional Connectivity

Whole-brain functional connectivity maps for the AI and for the PMC were generated for each subject. For the AI seed we used a dorsal AI seed derived from the parcellations used in a study on functional insular subdivisions (Deen et al., 2010). The PMC seed was derived from a previous study in our group on narrative processing, defined as a cluster in the posterior medial region that was more activated by reading sacred stories compared with non-sacred stories (Kaplan et al., 2017). The time-series for each of these ROI’s were extracted for each subject. These extracted time-series were then used as regressors for a first level general linear model analysis in FSL’s FEAT. Anatomical brain images for each subject were parcellated using FAST to generate a mask of cerebrospinal fluid (CSF) and the timecourse of signal from CSF was included as a nuisance regressor in this analysis. The resulting functional connectivity maps indicated how correlated any given voxel’s time series was with the time series of the seed region. Individual unthresholded functional connectivity maps were then subjected to the cross-subject MVPA analysis. Additionally, we performed a group-level GLM to describe connectivity with the seed regions during story listening overall. This group GLM modeled only the group level mean and used a FLAME mixed-effects model to generate group level statistical maps in the standard space which were then thresholded using FSL’s cluster thresholding algorithm with a cluster-forming threshold of Z > 3.1 and a cluster size probability of *p* < 0.05.

#### Multivariate Pattern Analysis

Searchlight multivariate pattern analysis (MVPA) (Kriegeskorte et al., 2006) was performed on the seed-connectivity maps using difference behavioral measures as the classifying attribute. Analyses were performed using PyMVPA (Hanke et al., 2009). For each analysis, subjects were divided into “high” or “low” categories for a variable based on whether they were above or below the mean on said variable.

The seed-connectivity maps for each story were classified for high vs. low reported transportation as measured by the TS-SF. Each story’s seed connectivity map was also classified based on the story-relevant prosocial measure (i.e., for “The Hitcher” the self-reported willingness to sign the hitchhiking dangers education petition) and the story-irrelevant prosocial measure (i.e., for “The Hitcher” the self-reported willingness to donate to cancer research). The story irrelevant prosocial measure analyses were included to distinguish whether the patterns of connectivity were reflecting a process of story-specific influence vs. capturing a more generic process related to general prosocial tendencies.

In the searchlight analyses, support-vector machine (SVM) classifiers were trained on the 5 mm spherical area surrounding each voxel using a leave-one-subject-out cross-validation approach. Each voxel in the resulting searchlight map thus represents the overall accuracy of the classifier in distinguishing high vs. low scores on that variable based upon seed-connectivity to the immediate area surrounding that voxel. The resulting maps indicate how accurate the classifier was at predicting the behavioral category based on data from each spatial location.

To determine an appropriate statistical thresholding, permutation testing was performed by repeatedly shuffling the map labels inside a single sphere and classifying the shuffled data to produce a distribution of accuracies under the null hypothesis. We then computed the number of resolution elements (resels) in the image by dividing the total volume of the brain by the volume of one sphere to yield the number of non-overlapping spheres. An alpha level of 0.05 was divided by the number of resels to determine a corrected alpha (Kaplan and Meyer, 2012). From this process, a threshold emerged of 75% accuracy for significance, which is the corrected *p*-value derived from the permutation test.

### Behavioral Results

The average score on the TS-SF relative to the cancer story (“The Living Room”) was 5.75 (SD = 1.07; ranging from 2.2 to 7.0) and relative to the hitchhiking story (“The Hitcher”) was 5.2 (SD = 1.28; ranging from 2.0 to 7.0). The transportation scores for the two stories were highly correlated within individuals (*r* = 0.511; *p* < 0.001), though subjects felt significantly more transported into the “Living Room” story compared to “The Hitcher” [*t*(36) = 2.82, *p* = 0.0078].

The average willingness to donate to cancer research after listening to the stories was 6.55 (SD = 3.04; ranging from 0.0–10.0) out of 10. The average willingness to sign a petition to educate kids on the dangers of hitchhiking was 6.55 (SD = 2.66; ranging from 0.1 to 10.0). These reported pro-social measures were significantly correlated within individuals (*r* = 0.417; *p* = 0.011); those who were more likely to support one measure were also more likely to support the other.

Narrative transportation in the cancer story was highly correlated with reported willingness to donate to cancer research (*r* = 0.425, *p* = 0.009). Narrative transportation in the cancer story was also correlated with willingness to sign the petition on the dangers of hitchhiking (*r* = 0.384, *p* = 0.02) (see Table 2). Narrative transportation in the hitchhiking story was not significantly correlated with either prosocial measure. This may be due to individuals feeling overall less transported into that story.

**TABLE 2:** Correlations between narrative transportation and pro-social intentions.

	Correlation Table		

		Transportation	

		Cancer Story	Hitchhiking Story
**Reported Willingness**	Cancer Donation	0.425**	0.228
	Hitchhiking Petition	0.384*	0.117
	*p*-value		
**Reported Willingness**	Cancer Donation	0.009	0.181
	Hitchhiking Petition	0.02	0.496

**p < 0.01; p < 0.05.

### Functional Connectivity: Group GLM

**FIGURE 1:**
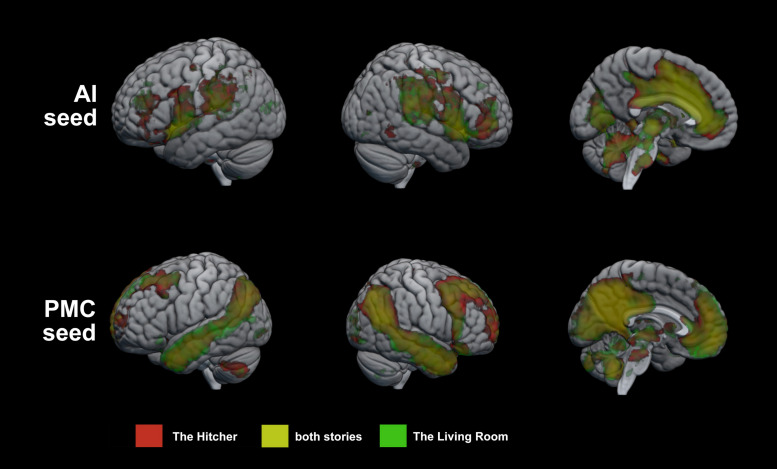
Group GLM of seed connectivity with AI and PMC. Regions of significant connectivity with the seed regions during story listening. Red = The Hitcher, Green = The Living Room, Yellow = connectivity with the seed during both stories.

Results of the group GLM of seed region connectivity are show in Figure 1. AI showed significant connectivity with large regions of the medial surface of the brain, including most of the cingulate gyrus and parts of the medial prefrontal cortex, the cuneus, the inferior parietal lobes, the precentral gyrus, the middle and inferior frontal gyri, and the cerebellum. PMC showed significant connectivity with DMN nodes, including medial prefrontal cortex, inferior parietal cortex, much of the temporal lobes. For both seeds, connectivity maps showed substantial overlap between the two stories.

### MVPA Searchlights: Narrative Transportation

For the cancer story, the mean split lead to 16 participants being classified as “low” in narrative transportation, and 20 being classified as “high.” For the hitchhiking story, the two groups were equally split with 18 participants each.

#### AI Connectivity Predictive of High vs. Low Transportation

**FIGURE 2:**
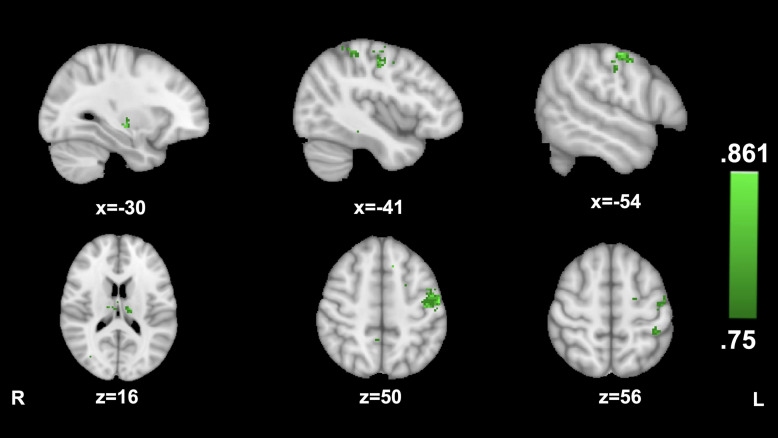
Anterior insula connectivity and narrative transportation during The Hitcher. MVPA searchlight showing above chance classification of AI connectivity maps for high vs. low transportation during the Hitchhiking story.

No clusters of AI connectivity significantly predicted transportation during the cancer story. Transportation during the hitchhiking story was significantly predicted by AI connectivity to a large cluster in the left precentral gyrus (−54, −12, and 50; 86.1%) as well as to smaller clusters in left postcentral gyrus (−41,−38, and 56; 80.5%) the left thalamus (−12, −17, and 16; 83.3%), and the left putamen (−30, −15, and −5; 83.3%) (see Figure 2).

#### PMC Connectivity Predictive of High vs. Low Transportation

Transportation during the cancer story was significantly predicted by PMC connectivity to clusters in left inferior frontal gyrus (−35, 15, and 20; 86.1%), right temporal pole (20, −19, and −32; 83.3%), the left thalamus (−8, −13, and −2; 86.1%), and left postcentral gyrus (−8, −36, and 75; 83.3%).

**FIGURE 3:**
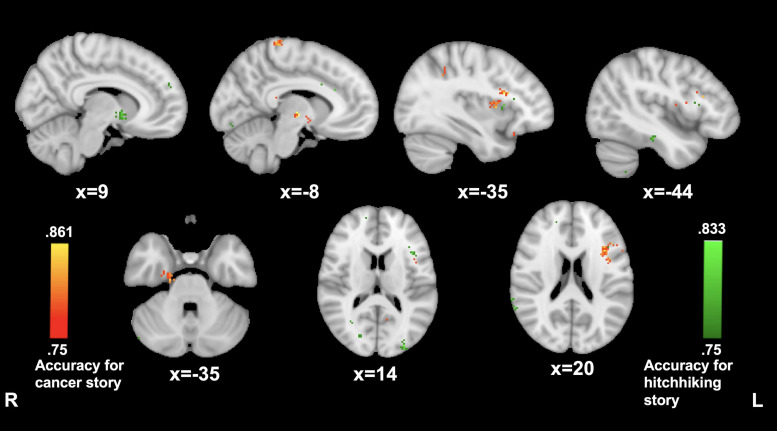
PMC connectivity predictive of narrative transportation. MVPA searchlights showing above chance classification of PMC connectivity maps for high vs. low transportation during The Hitcher (green), and The Living Room (red).

Transportation during the hitchhiking story was significantly predicted by PMC connectivity with a cluster in the right caudate (9, 6, −3; 80.5%), as well as several smaller clusters in left inferior frontal gyrus (−44, 16, and 16; 80.5%), left inferior temporal cortex (−44, −26, and −20; 80.5%), and the left visual-association area (−30, −90, and 14; 83.3%) (see Figure 3).

### MVPA Searchlights: Prosocial Intention

For willingness to donate to cancer research, the mean split lead to 20 subjects being classified as “low” willingness and 16 as “high.” For willingness to donate to hitchhiking dangers awareness, 15 were classified as “low” and 21 were classified as “high.”

#### AI Connectivity Predictive of High vs. Low Prosocial Intentions

##### Cancer Story

**FIGURE 4:**
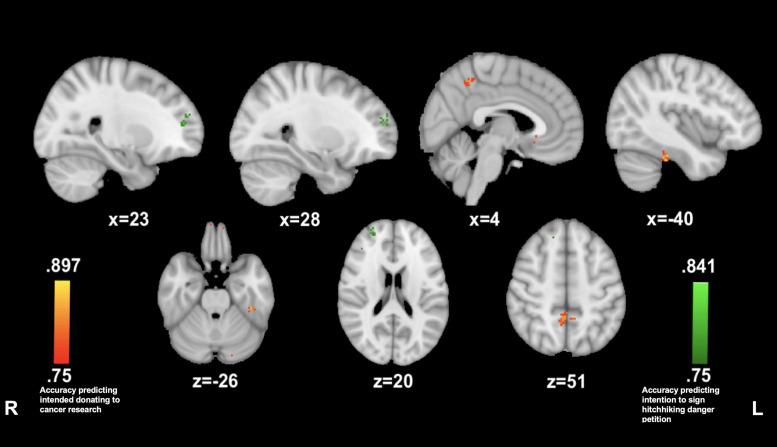
Anterior insula connectivity predictive of prosocial intention during *The Living Room.* MVPA searchlights showing above chance classification of AI connectivity maps for high vs. low prosocial intention for related (red) vs. unrelated (green) issues during The Living Room.

Reported willingness to donate to cancer research was predicted by insula connectivity with small clusters in the precuneus (4, −47, and 50; 83.3%) and in the left posterior fusiform gyrus (−40, −32, and −27; 86.1%) while listening to the cancer story. The reported willingness to sign the petition on hitchhiking dangers awareness was predicted by insula connectivity with a cluster in the right anterior prefrontal cortex (23, 50, and 14; 83.3%) (see Figure 4).

##### Hitchhiking Story

**FIGURE 5:**
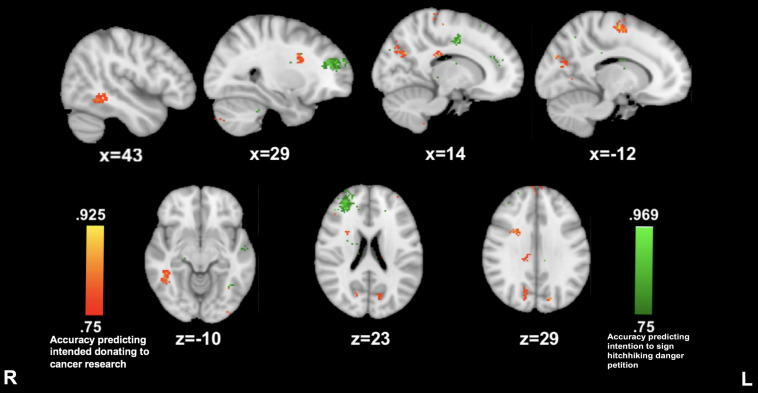
Anterior insula connectivity predictive of prosocial intention during *The Hitcher*. MVPA searchlights showing above chance classification of AI connectivity maps for high vs. low prosocial intention for related (red) vs. unrelated (green) issues during The Hitcher.

Reported willingness to sign the petition on hitchhiking dangers awareness was predicted by a prominent cluster of insula connectivity to the right anterior prefrontal cortex (29, 45, and 20; 86.1%) while listening to the hitchhiking story. The reported willingness to donate to cancer research was predicted by insula connectivity to clusters in left precentral gyrus (−12, −10, and 62; 91.6%) precuneus (13, −66, and 28; 88.8%) and right superior temporal gyrus (43, −48, and −9; 83.3%) (see Figure 5).

#### PMC Connectivity Predictive of High vs. Low Prosociality

##### Cancer Story

**FIGURE 6:**
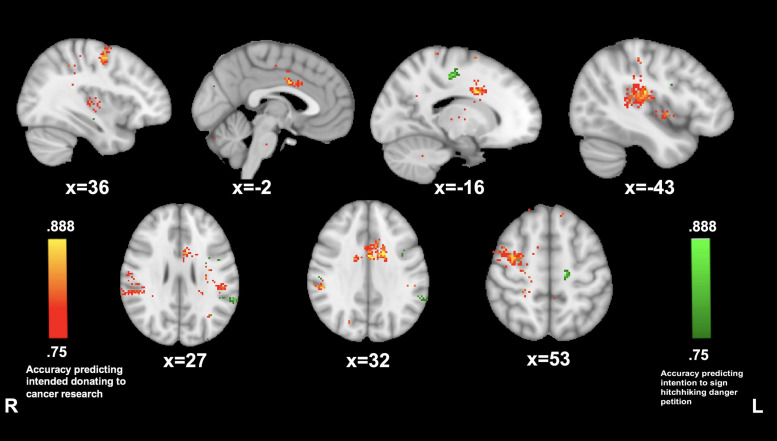
Posterior medial cortex connectivity predictive of prosocial intention during *The Living Room*. MVPA searchlights showing above chance classification of PMC connectivity maps for high vs. low prosocial intention for related (red) vs. unrelated (green) issues during The Living Room.

Reported willingness to donate to cancer was predicted by PMC connectivity with left postcentral gyrus (−43, −22, and 20; 88.8%), the anterior cingulate (−2, 3, 32; 88.8%), right supramarginal gyrus (53, −34, and 32; 86.1%), and right supplementary motor area (36, −4, and 53; 86.1%). Reported willingness to sign the petition on hitchhiking dangers awareness was predicted by PMC connectivity to the left premotor area (−16, −20, and 49; 88.8%) and to the left angular gyrus (−62, −44, and 27; 83.3%) (see Figure 6).

##### Hitchhiking Story

PMC connectivity while listening to the hitchhiking story was neither significantly predictive of willingness to donate to cancer research nor willingness to sign a hitchhiking dangers petition.

## Discussion

Our hypothesis that patterns of functional connectivity would be predictive of behavioral characteristics was largely supported. Patterns of AI connectivity significantly predicted the intention to support to both causes during both stories. AI connectivity, however, was only predictive of narrative transportation during the hitchhiking story. Patterns of PMC functional connectivity were only predictive of the intention to support causes during the cancer story. However, PMC functional connectivity was predictive of narrative transportation for both stories. These data demonstrate that there are different patterns of functional connectivity while listening to stories between those who feel transported and those who do not. Likewise, there are different patterns of functional connectivity between those who intend to support a cause and those who do not. The predictive patterns of connectivity in these analyses did correspond to some DMN and sensorimotor regions as predicted (such as angular gyrus, the precuneus, precentral gyrus, and postcentral gyrus).

More ambitiously, we hypothesized that patterns of connectivity to DMN and sensorimotor areas would be consistent across both stories. This consistency would demonstrate that the underlying processes were general to narratives rather than specific to one story or the other. For the analyses involving narrative transportation, this hypothesis would have been borne out if the seed regions were significantly connected with the same clusters in both stories. For the most part this was not supported by the data. Despite similar patterns of functional connectivity while listening to both stories, there was only one area of possible overlap for the two stories in predicting narrative transportation: PMC connectivity with left inferior frontal gyrus (IFG). This region was not one we had predicted, and will be further discussed.

For the analyses predicting prosocial intentions, we hypothesized that connectivity from the seed region would be the same for the cancer story predicting intention to donate to cancer research as it would be for the hitchhiking story predicting intention to sign the hitchhiking danger petition. In short, this would demonstrate functional connectivity consistently corresponding to a story-specific influence. This was not the case. Furthermore, we found that functional connectivity during stories were predictive of the intention to support causes that were irrelevant to its content. The lack of a story-specific effect suggests that these patterns of functional connectivity were not directly related to the stories’ influence persuading people on specific causes.

While these results did not suggest a unified process across narrative transportation and persuasion, these data may show that these processes are specific to story content. The specific content of a story may influence which processes play a role in transportation or in eliciting empathetic responses. For example: anecdotal reports from listeners suggest that the hitchhiking story was less emotionally compelling throughout most of its length. Long periods without emotionally gripping content may make the story simply a worse stimulus for studying narratives’ effects on persuading prosocial responses. Furthermore, unique elements of the hitchhiking story, such as more physical action, may explain why its AI connectivity patterns could successfully classify subjects based on narrative transportation. Notably, subjects reported being more transported into the Living Room story. This difference in transportation between the two stories may contribute to differences in how connectivity patterns are predictive of prosocial causes.

The differences in the functional connectivity patterns underlying transportation and intention to support causes between the two stories highlights a challenge for fMRI studies that employ naturalistic stimuli. There is a necessary tradeoff in studies using this design between ecological validity and experimental control. Because stories, movies, and musical pieces that aren’t designed by experimenters will necessarily differ from each other in content, emotional dynamics, and many other characteristics, results obtained with a single stimulus are less likely to generalize to others. The same qualities that make these media compelling as stimuli make them unique. One lesson we take from our results, therefore, is to stress the need for analyzing multiple different naturalistic stimuli in order to draw generalizable conclusions.

### PMC-IFG Connectivity Across Both Stories

The one consistent pattern of connectivity found between both stories was connectivity between the PMC and the IFG predicting high vs. low transportation. The left IFG and PMC have been shown to work in tandem for a variety of tasks. For example, when listening to music the left IFG is believe to play a role in perceiving emotion in the musical structure whereas the PMC is involved in feeling those emotions (Tabei, 2015). The left IFG and PMC also work together for recognition memory of newly encoded musical stimuli (Watanabe et al., 2008) and pictures (Lundstrom et al., 2005). There are obvious parallels between these processes and those believed to be at play in narrative processing. One of the cognitive processes which makes narrative transportation possible is perceiving emotions and contexts within stimuli which relate to autobiographical memories (Baumgartner et al., 1992; Hartmann et al., 2016). The perception of these elements in narratives may trigger an involuntary retrieval of autobiographical memories (Moore and Homer, 2008; McDonald et al., 2015). Like with music and pictures, the IFG may parse the emotional and content-related cues in narratives before PMC regions relate them to memory traces. The linguistic nature of stories may be further evidence of this pathways role. The left IFG is known to play a role in parsing the semantic units of sentences, such as where the verb or subject is located within a sentence. The precuneus may then “store” these units to construct an overall meaning (Chou et al., 2009; Obleser and Kotz, 2010; Meyer et al., 2012). The use of this pathway in the parsing semantic meaning coupled with the PMC’s role in autobiographical memory and the self may explain this connection between narrative comprehension and the self (Regev et al., 2013). In fact, the left IFG has been found to be active in 55.9% of all studies focusing on self-referential processes (Morin and Michaud, 2007). It is believed this represents the crucial role of inner-speech in self-referential processes. This idea mirrors the theory that autobiographical knowledge is organized in a narrative manner, demonstrating how linked these linguistic forms of semantic ordering are to the self (Robinson and Taylor, 1998; Singer and Bluck, 2001; Conway et al., 2004).

### AI Connectivity During the Hitchhiking Story Predicts Transportation

For the hitchhiking story, AI connectivity was found to be predictive of narrative transportation. The regions where AI connectivity and was predictive of narrative transportation (precentral gyrus, postcentral gyrus, the thalamus, and the putamen) appear to tell a unified story. These regions may suggest a link with sensory and motor imagery (Szameitat et al., 2007; Belardinelli et al., 2009). Interestingly, it is the posterior insula not the AI which is normally associated with functional connectivity to sensorimotor regions (Cauda et al., 2011). However, some data suggests that the AI may play a role in integrating sensorimotor information with affective information (Kurth et al., 2010; Kleber et al., 2017). The hitchhiking story at its climax involves the protagonists making a run away from their potential captors: an element of physicality not present in the cancer story. It may be this sensorimotor imagery which creates the strongest link for immersion into the hitchhiking story. Those who can more effectively simulate these actions have more functional connectivity between the AI and sensorimotor regions, and are more likely to feel as if they are truly within the story. This would be a different cognitive process facilitating narrative transportation for this story as compared to the cancer story.

### PMC Connectivity During the Cancer Story Was Predictive of Prosocial Behaviors

The patterns of PMC connectivity which predicted the intention to donate to cancer research while listening to the story may relate to listeners’ empathy with the characters in this emotionally evocative story. The anterior cingulate, supplementary motor area, postcentral gyrus, and supramarginal gyrus have all been found to associate with empathy for pain (Lloyd et al., 2004; Lawrence et al., 2006; Morelli et al., 2014). Furthermore, the activity and structural integrity of these regions have been found to associate with reported intention for, and real-world, prosocial behaviors (Hayashi et al., 2014; Morelli et al., 2014). The cancer story involved vivid discussions of watching somebody get sicker and sicker. It is likely that more vividly somebody experiences this character’s suffering the more they empathize, and thus the more the narrative influences their intention to donate to cancer research. The right supramarginal gyrus may play an important role in this process. This region is believed to be involved in determining the emotional “reference point” through which judgments are made; simply put: whether or not the person of reference is the self or another person (Hayashi et al., 2014; Hoffmann et al., 2016). While the supramarginal gyrus is under normal circumstances more active for self-reference than for referencing others (Vogeley and Fink, 2003; van der Heiden et al., 2013), it has been found that heightened activity in the region associates with using a third-party as the emotional point of reference (Silani et al., 2013; Steinbeis and Singer, 2014; Riva et al., 2016). PMC connectivity to this region may represent this “switching” mechanism, with the emotional information processed from the story now being utilized as the new reference point. This would also explain why motor and sensory regions were also experiencing connectivity with the PMC, simulating the physical experience of the character.

### Prosocial Tendencies as an Individual Trait

While we hypothesized that pro-social intentions would only be predicted in a story-specific manner (i.e., the cancer story would predict willingness to donate to cancer and the hitchhiking story would predict willingness to donate to hitchhiking danger awareness), this was not the case. AI connectivity was predictive of pro-social intentions for **both** causes during both stories, and PMC connectivity was predictive of pro-social intentions for both causes during *The Living Room.* Functional connectivity during different stories therefore does not appear to be able to disentangle personal characteristics from story-specific influence in this case. It is possible that these patterns of connectivity are related to the manner in which individuals engage with all narratives regardless of the specific context. Pro-social giving was significantly correlated across questions; in other words, people who indicated prosocial intent for one scenario were more likely to indicate high prosocial intent for the other as well. The trait underlying this willingness to give may be marked by specific patterns of functional connectivity. For example, AI connectivity to the right anterior prefrontal cortex was significantly predictive of willingness to donate to the hitchhiking dangers petition during both stories. Connectivity between the AI and prefrontal cortex has been previously implicated in the regulation of empathic responses (Pino et al., 2016; Yao et al., 2016). This type of individual ability could be evident during narrative processing and relate to the tendency to give regardless of the specific context of the narrative.

## Conclusion

We found that individual differences in the subjective immersion into a narrative world, and the intention to contribute to a cause related to the narrative can be accurately predicted from functional connectivity while listening to the story. Our more specific hypotheses on what these patterns of predictive connectivity would look like focused on what processes may be alike between the two stories. The results instead reveal a more valuable question: how does a story’s specific content influence the cognitive and affective functions used to process it? With the diversity of cognitive processes thought to be involved narrative transportation (empathy, autobiographical memory, perspective taking, mental imagery etc.) different stories are bound to be facilitated differentially by different processes. This underscores the necessity of employing multiple naturalistic stimuli that differ in content when studying generalized processes like transportation and empathy, to support generalization of results.

## Data Availability Statement

The raw data supporting the conclusions of this article will be made available by the authors, without undue reservation.

## Ethics Statement

The studies involving human participants were reviewed and approved by USC Institutional Review Board. The patients/participants provided their written informed consent to participate in this study.

## Author Contributions

SG helped to design the study and collect the data. BS participated in the data analysis. AV participated in the data analysis and writing the manuscript. JK participated in the study design, data collection, analysis, and manuscript preparation. All authors contributed to the article and approved the submitted version.

## Conflict of Interest

The authors declare that the research was conducted in the absence of any commercial or financial relationships that could be construed as a potential conflict of interest.

## Footnotes

1 http://fsl.fmrib.ox.ac.uk/fsl/fslwiki/

## References

[B1] Appel M. Gnambs T. Richter T. Green M. C. (2015). The transportation scale–short form (TS–SF). *Media Psychol.* 18 243–266. 10.1080/15213269.2014.987400

[B2] Appel M. Richter T. (2010). Transportation and need for affect in narrative persuasion: a mediated moderation model. *Media Psychol.* 13 101–135. 10.1080/15213261003799847

[B3] Araujo H. F. Kaplan J. Damasio H. Damasio A. (2015). Neural correlates of different self domains. *Brain Behav.* 5:e00409. 10.1002/brb3.409 26807336 PMC4714646

[B4] Baldassano C. Chen J. Zadbood A. Pillow J. W. Hasson U. Norman K. A. (2017). Discovering event structure in continuous narrative perception and memory. *Neuron* 95 709–721.e5. 10.1016/j.neuron.2017.06.041 28772125 PMC5558154

[B5] Baldassano C. Hasson U. Norman K. A. (2018). Representation of real-world event schemas during narrative perception. *J. Neurosci.* 38 9689–9699. 10.1523/JNEUROSCI.0251-18.2018 30249790 PMC6222059

[B6] Banerjee S. C. Greene K. (2012). Role of transportation in the persuasion process: cognitive and affective responses to Antidrug narratives. *J. Health Commun.* 17 564–581. 10.1080/10810730.2011.635779 22475073

[B7] Batat W. Wohlfeil M. (2009). Getting lost ‘Into the Wild’: understanding consumers’ movie enjoyment through a narrative transportation approach. *Adv. Consum. Res.* 36 372–377.

[B8] Baumgartner H. Sujan M. Bettman J. R. (1992). Autobiographical memories, affect, and consumer information processing. *J. Consum. Psychol.* 1 53–82. 10.1207/s15327663jcp0101_04

[B9] Belardinelli M. O. Palmiero M. Sestieri C. Nardo D. Di Matteo R. Londei A. (2009). An fMRI investigation on image generation in different sensory modalities: the influence of vividness. *Acta Psychol.* 132 190–200. 10.1016/j.actpsy.2009.06.009 19695558

[B10] Bilandzic H. Busselle R. (2013). “Narrative persuasion,” in *The Sage Handbook of Persuasion: Developments in Theory and Practice*, 2nd Edn, eds Dillard J. P. Shen L. (Thousand Oaks, CA: Sage), 200–219. 10.4135/9781452218410.n13

[B11] Breen A. V. McLean K. C. Cairney K. McAdams D. P. (2017). Movies, books, and identity: exploring the narrative ecology of the self. *Qual. Psychol.* 4 243–259. 10.1037/qup0000059

[B12] Bruneau E. Dufour N. Saxe R. (2013). How we know it hurts: item analysis of written narratives reveals distinct neural responses to others’ physical pain and emotional suffering. *PLoS One* 8:e63085. 10.1371/journal.pone.0063085 23638181 PMC3637309

[B13] Caputo N. M. Rouner D. (2011). Narrative processing of entertainment media and mental illness stigma. *Health Commun.* 26 595–604. 10.1080/10410236.2011.560787 21516556

[B14] Cauda F. D’Agata F. Sacco K. Duca S. Geminiani G. Vercelli A. (2011). Functional connectivity of the insula in the resting brain. *Neuroimage* 55 8–23. 10.1016/j.neuroimage.2010.11.049 21111053

[B15] Chou T.-L. Chen C.-W. Wu M.-Y. Booth J. R. (2009). The role of inferior frontal gyrus and inferior parietal lobule in semantic processing of Chinese characters. *Exp. Brain Res.* 198 465–475. 10.1007/s00221-009-1942-y 19618170 PMC3277261

[B16] Christy K. R. Fox J. (2016). Transportability and presence as predictors of avatar identification within narrative video games. *Cyberpsychol. Behav. Soc. Netw.* 19 283–287. 10.1089/cyber.2015.0474 26919032

[B17] Conway M. A. Singer J. A. Tagini A. (2004). The self and autobiographical memory: correspondence and coherence. *Soc. Cogn.* 22 491–529. 10.1521/soco.22.5.491.50768

[B18] Craig A. D. (2009). How do you feel–now? The anterior insula and human awareness. *Nat. Rev. Neurosci.* 10 59–70. 10.1038/nrn2555 19096369

[B19] Deen B. Pitskel N. B. Pelphrey K. A. (2010). Three systems of insular functional connectivity identified with cluster analysis. *Cereb. Cortex* 21 1498–1506. 10.1093/cercor/bhq186 21097516 PMC3116731

[B20] Dehghani M. Boghrati R. Man K. Hoover J. Gimbel S. I. Vaswani A. (2017). Decoding the neural representation of story meanings across languages. *Hum. Brain Mapp.* 38 6096–6106. 10.1002/hbm.23814 28940969 PMC6867091

[B21] Deighton J. Romer D. McQueen J. (1989). Using drama to persuade. *J. Consum. Res.* 16 335–343. 10.1086/209219

[B22] Dillard A. J. Ferrer R. A. Welch J. D. (2018). Associations between narrative transportation, risk perception and behaviour intentions following narrative messages about skin cancer. *Psychol. Health* 33 573–593. 10.1080/08870446.2017.1380811 28975805

[B23] Dunlop S. M. Wakefield M. Kashima Y. (2010). Pathways to persuasion: cognitive and experiential responses to health-promoting mass media messages. *Commun. Res.* 37 133–164. 10.1177/0093650209351912

[B24] Escalas J. E. (2004). Imagine yourself in the product: mental simulation, narrative transportation, and persuasion. *J. Advert.* 33 37–48. 10.1080/00913367.2004.10639163

[B25] Feng C. Yan X. Huang W. Han S. Ma Y. (2018). Neural representations of the multidimensional self in the cortical midline structures. *Neuroimage* 183 291–299. 10.1016/j.neuroimage.2018.08.018 30118871

[B26] Fivush R. (2011). The development of autobiographical memory. *Annu. Rev. Psychol.* 62 559–582. 20636128 10.1146/annurev.psych.121208.131702

[B27] Fransson P. Marrelec G. (2008). The precuneus/posterior cingulate cortex plays a pivotal role in the default mode network: evidence from a partial correlation network analysis. *Neuroimage* 42 1178–1184. 10.1016/j.neuroimage.2008.05.059 18598773

[B28] Green M. C. Brock T. C. (2000). The role of transportation in the persuasiveness of public narratives. *J. Pers. Soc. Psychol.* 79 701–721. 10.1037/0022-3514.79.5.701 11079236

[B29] Green M. C. Brock T. C. Kaufman G. F. (2004). Understanding media enjoyment: the role of transportation into narrative worlds. *Commun. Theory* 14 311–327. 10.1111/j.1468-2885.2004.tb00317.x

[B30] Green M. C. Chatham C. Sestir M. A. (2012). Emotion and transportation into fact and fiction. *Sci. Study Lit.* 2 37–59. 10.1075/ssol.2.1.03gre 33486653

[B31] Green M. C. Clark J. L. (2013). Transportation into narrative worlds: implications for entertainment media influences on tobacco use. *Addiction* 108 477–484. 10.1111/j.1360-0443.2012.04088.x 22994374

[B32] Green M. C. Donahue J. K. (2009). “Simulated worlds: transportation into narratives,” in *Handbook of Imagination and Mental Simulation*, eds Markman K. Klein W. M. Suhr J. A. (New York, NY: Psychology Press) 241–256.

[B33] Green M. C. Sestir M. (2017). *Transportation Theory. The International Encyclopedia of Media Effects* (Hoboken, NJ: John Wiley and Sons, Inc), 1–14. 10.1002/9781118783764.wbieme0083

[B34] Greenwood D. N. Long C. R. (2009). Psychological predictors of media involvement: solitude experiences and the need to belong. *Commun. Res.* 36 637–654. 10.1177/0093650209338906

[B35] Gu X. Hof P. R. Friston K. J. Fan J. (2013). Anterior insular cortex and emotional awareness. *J. Comp. Neurol.* 521 3371–3388. 10.1002/cne.23368 23749500 PMC3999437

[B36] Guerrero I. Igartua J. J. (2017). “The role of the narrator point of view and the similarity with the protagonist in narratives designed to reduce prejudice towards stigmatized immigrants,” in *Proceedings of the 5th International Conference on Technological Ecosystems for Enhancing Multiculturality*, Vol. 94 (New York, NY), 1–5. 10.1145/3144826.3145441

[B37] Hall A. E. Bracken C. C. (2011). I really liked that movie. Testing the relationship between trait empathy, transportation, perceived realism, and movie enjoyment. *J. Media Psychol.* 23 90–99. 10.1027/1864-1105/a000036

[B38] Hanke M. Halchenko Y. O. Sederberg P. B. Hanson S. J. Haxby J. V. Pollmann S. (2009). PyMVPA: a python toolbox for multivariate pattern analysis of fMRI data. *Neuroinformatics* 7 37–53. 10.1007/s12021-008-9041-y 19184561 PMC2664559

[B39] Harjusola-Webb S. Hubbell S. P. Bedesem P. (2012). Increasing prosocial behaviors of young children with disabilities in inclusive classrooms using a combination of peer-mediated intervention and social narratives. *Beyond Behav.* 21 29–36.

[B40] Hartmann P. Apaolaza V. Eisend M. (2016). Nature imagery in non-green advertising: the effects of emotion, autobiographical memory, and consumer’s green traits. *J. Advert.* 45 427–440. 10.1080/00913367.2016.1190259

[B41] Hasson U. Furman O. Clark D. Dudai Y. Davachi L. (2008). Enhanced intersubject correlations during movie viewing correlate with successful episodic encoding. *Neuron* 57 452–462. 10.1016/j.neuron.2007.12.009 18255037 PMC2789242

[B42] Hayashi A. Abe N. Fujii T. Ito A. Ueno A. Koseki Y. (2014). Dissociable neural systems for moral judgment of anti-and pro-social lying. *Brain Res.* 1556 46–56. 10.1016/j.brainres.2014.02.011 24530270

[B43] Hirschman E. C. (1983). Predictors of self-projection, fantasy fulfillment, and escapism. *J. Soc. Psychol.* 120 63–76. 10.1080/00224545.1983.9712011

[B44] Hoffmann F. Koehne S. Steinbeis N. Dziobek I. Singer T. (2016). Preserved self-other distinction during empathy in autism is linked to network integrity of right supramarginal gyrus. *J. Autism. Dev. Disord.* 46 637–648. 10.1007/s10803-015-2609-0 26476740

[B45] Igartua J.-J. Frutos F. J. (2017). Enhancing attitudes toward stigmatized groups with movies: mediating and moderating processes of narrative persuasion. *Int. J. Commun.* 11 158–177.

[B46] Immordino-Yang M. H. McColl A. Damasio H. Damasio A. (2009). Neural correlates of admiration and compassion. *Proc. Natl. Acad. Sci. U.S.A.* 106 8021–8026. 10.1073/pnas.0810363106 19414310 PMC2670880

[B47] Immordino-Yang M. H. Yang X.-F. Damasio H. (2014). Correlations between social-emotional feelings and anterior insula activity are independent from visceral states but influenced by culture. *Front. Hum. Neurosci.* 8:728. 10.3389/fnhum.2014.00728 25278862 PMC4165215

[B48] Immordino-Yang M. H. Yang X.-F. Damasio H. (2016). Cultural modes of expressing emotions influence how emotions are experienced. *Emotion* 16 1033–1039. 10.1037/emo0000201 27270077 PMC5042821

[B49] Jabbi M. Bastiaansen J. Keysers C. (2008). A common anterior insula representation of disgust observation, experience and imagination shows divergent functional connectivity pathways. *PLoS One* 3:e2939. 10.1371/journal.pone.0002939 18698355 PMC2491556

[B50] Johnson D. R. (2012). Transportation into a story increases empathy, prosocial behavior, and perceptual bias toward fearful expressions. *Pers. Individ. Dif.* 52 150–155. 10.1016/j.paid.2011.10.005

[B51] Johnson D. R. Huffman B. L. Jasper D. M. (2014). Changing race boundary perception by reading narrative fiction. *Basic Appl. Soc. Psychol.* 36 83–90. 10.1080/01973533.2013.856791

[B52] Johnson D. R. Jasper D. M. Griffin S. Huffman B. L. (2013). Reading narrative fiction reduces Arab-Muslim prejudice and offers a safe haven from intergroup anxiety. *Soc. Cogn.* 31 578–598. 10.1521/soco.2013.31.5.578

[B53] Kaplan J. Gimbel S. I. Dehghani M. Immordino-Yang M. H. Sagae K. Wong J. D. (2017). Processing narratives concerning protected values: a cross-cultural investigation of neural correlates. *Cereb. Cortex* 27 1428–1438. 26744541 10.1093/cercor/bhv325

[B54] Kaplan J. Meyer K. (2012). Multivariate pattern analysis reveals common neural patterns across individuals during touch observation. *Neuroimage* 60 204–212. 10.1016/j.neuroimage.2011.12.059 22227887 PMC3313627

[B55] Khalsa S. Mayhew S. D. Chechlacz M. Bagary M. Bagshaw A. P. (2014). The structural and functional connectivity of the posterior cingulate cortex: comparison between deterministic and probabilistic tractography for the investigation of structure–function relationships. *Neuroimage* 102 118–127. 10.1016/j.neuroimage.2013.12.022 24365673

[B56] Kleber B. Friberg A. Zeitouni A. Zatorre R. (2017). Experience-dependent modulation of right anterior insula and sensorimotor regions as a function of noise-masked auditory feedback in singers and nonsingers. *Neuroimage* 147 97–110. 10.1016/j.neuroimage.2016.11.059 27916664

[B57] Klimmt C. Hefner D. Vorderer P. (2009). The video game experience as “true” identification: a theory of enjoyable alterations of players’ self-perception. *Commun. Theory* 19 351–373. 10.1111/j.1468-2885.2009.01347.x

[B58] Kriegeskorte N. Bandettini P. (2007). Analyzing for information, not activation, to exploit high-resolution fMRI. *Neuroimage* 38 649–662. 10.1016/j.neuroimage.2007.02.022 17804260 PMC2099257

[B59] Kriegeskorte N. Goebel R. Bandettini P. (2006). Information-based functional brain mapping. *Proc. Natl. Acad. Sci. U.S.A.* 103 3863–3868. 10.1073/pnas.0600244103 16537458 PMC1383651

[B60] Kuo A. Lutz R. J. Hiler J. L. (2016). Brave new World of Warcraft: a conceptual framework for active escapism. *J. Consum. Mark.* 33 498–506. 10.1108/jcm-04-2016-1775

[B61] Kurth F. Zilles K. Fox P. T. Laird A. R. Eickhoff S. B. (2010). A link between the systems: functional differentiation and integration within the human insula revealed by meta-analysis. *Brain Struct. Funct.* 214 519–534. 10.1007/s00429-010-0255-z 20512376 PMC4801482

[B62] Larkey L. K. Hecht M. (2010). A model of effects of narrative as culture-centric health promotion. *J. Health Commun.* 15 114–135. 10.1080/10810730903528017 20390982

[B63] Lawrence E. J. Shaw P. Giampietro V. Surguladze S. Brammer M. J. David A. S. (2006). The role of ‘shared representations’ in social perception and empathy: an fMRI study. *Neuroimage* 29 1173–1184. 10.1016/j.neuroimage.2005.09.001 16337816

[B64] Lerner Y. Honey C. J. Silbert L. J. Hasson U. (2011). Topographic mapping of a hierarchy of temporal receptive windows using a narrated story. *J. Neurosci.* 31 2906–2915. 10.1523/JNEUROSCI.3684-10.2011 21414912 PMC3089381

[B65] Li D. Liau A. Khoo A. (2011). Examining the influence of actual-ideal self-discrepancies, depression, and escapism, on pathological gaming among massively multiplayer online adolescent gamers. *Cyberpsychol. Behav. Soc. Netw.* 14 535–539. 10.1089/cyber.2010.0463 21332374

[B66] Lloyd D. Di Pellegrino G. Roberts N. (2004). Vicarious responses to pain in anterior cingulate cortex: is empathy a multisensory issue? *Cogn. Affect. Behav. Neurosci.* 4 270–278. 10.3758/cabn.4.2.270 15460933

[B67] Lundstrom B. N. Ingvar M. Petersson K. M. (2005). The role of precuneus and left inferior frontal cortex during source memory episodic retrieval. *Neuroimage* 27 824–834. 10.1016/j.neuroimage.2005.05.008 15982902

[B68] Mazzocco P. J. Green M. C. Sasota J. A. Jones N. W. (2010). This story is not for everyone: transportability and narrative persuasion. *Soc. Psychol. Pers. Sci.* 1 361–368. 10.1177/1948550610376600

[B69] McDonald D. G. Sarge M. A. Lin S.-F. Collier J. G. Potocki B. (2015). A role for the self: media content as triggers for involuntary autobiographical memories. *Commun. Res.* 42 3–29. 10.1177/0093650212464771

[B70] Meyer L. Obleser J. Anwander A. Friederici A. D. (2012). Linking ordering in Broca’s area to storage in left temporo-parietal regions: the case of sentence processing. *Neuroimage* 62 1987–1998. 10.1016/j.neuroimage.2012.05.052 22634860

[B71] Moore D. J. Homer P. M. (2008). Self-brand connections: the role of attitude strength and autobiographical memory primes. *J. Bus. Res.* 61 707–714. 10.1016/j.jbusres.2007.09.002

[B72] Morelli S. A. Rameson L. T. Lieberman M. D. (2014). The neural components of empathy: predicting daily prosocial behavior. *Soc. Cogn. Affect. Neurosci.* 9 39–47. 10.1093/scan/nss088 22887480 PMC3871722

[B73] Morin A. Michaud J. (2007). Self-awareness and the left inferior frontal gyrus: inner speech use during self-related processing. *Brain Res. Bull.* 74 387–396. 10.1016/j.brainresbull.2007.06.013 17920447

[B74] Morris B. S. Chrysochou P. Christensen J. D. Orquin J. L. Barraza J. Zak P. J. (2019). Stories vs. facts: triggering emotion and action-taking on climate change. *Clim. Change* 154 19–36. 10.1007/s10584-019-02425-6

[B75] Moyer-Gusé E. Dale K. R. Ortiz M. (2019). Reducing prejudice through narratives: an examination of the mechanisms of vicarious intergroup contact. *J. Media Psychol.* 31 185–195. 10.1027/1864-1105/a000249

[B76] Nguyen M. Vanderwal T. Hasson U. (2019). Shared understanding of narratives is correlated with shared neural responses. *Neuroimage* 184 161–170. 10.1016/j.neuroimage.2018.09.010 30217543 PMC6287615

[B77] Nguyen V. T. Breakspear M. Hu X. Guo C. C. (2016). The integration of the internal and external milieu in the insula during dynamic emotional experiences. *Neuroimage* 124 455–463. 10.1016/j.neuroimage.2015.08.078 26375211

[B78] Nomi J. S. Farrant K. Damaraju E. Rachakonda S. Calhoun V. D. Uddin L. Q. (2016). Dynamic functional network connectivity reveals unique and overlapping profiles of insula subdivisions. *Hum. Brain Mapp.* 37 1770–1787. 10.1002/hbm.23135 26880689 PMC4837017

[B79] Norman K. A. Polyn S. M. Detre G. J. Haxby J. V. (2006). Beyond mind-reading: multi-voxel pattern analysis of fMRI data. *Trends Cogn. Sci.* 10 424–430. 10.1016/j.tics.2006.07.005 16899397

[B80] Obleser J. Kotz S. A. (2010). Expectancy constraints in degraded speech modulate the language comprehension network. *Cereb. Cortex* 20 633–640. 10.1093/cercor/bhp128 19561061

[B81] Parvizi J. Van Hoesen G. W. Buckwalter J. Damasio A. (2006). Neural connections of the posteromedial cortex in the macaque. *Proc. Natl. Acad. Sci. U.S.A.* 103 1563–1568. 10.1073/pnas.0507729103 16432221 PMC1345704

[B82] Pino M. C. Tempesta D. Catalucci A. Anselmi M. Nigri A. Iaria G. (2016). Altered cortico-limbic functional connectivity during an empathy task in subjects with post-traumatic stress disorder. *J. Psychopathol. Behav. Assess.* 38 398–405. 10.1007/s10862-016-9538-x

[B83] Prebble S. C. Addis D. R. Tippett L. J. (2013). Autobiographical memory and sense of self. *Psychol. Bull.* 139 815–840. 10.1037/a0030146 23025923

[B84] Rameson L. T. Satpute A. B. Lieberman M. D. (2010). The neural correlates of implicit and explicit self-relevant processing. *Neuroimage* 50 701–708. 10.1016/j.neuroimage.2009.12.098 20045472

[B85] Rasmussen A. S. Johannessen K. B. Berntsen D. (2014). Ways of sampling voluntary and involuntary autobiographical memories in daily life. *Conscious. Cogn.* 30 156–168. 10.1016/j.concog.2014.09.008 25299944

[B86] Regev M. Honey C. J. Simony E. Hasson U. (2013). Selective and invariant neural responses to spoken and written narratives. *J. Neurosci.* 33 15978–15988. 10.1523/jneurosci.1580-13.2013 24089502 PMC3787506

[B87] Riva F. Triscoli C. Lamm C. Carnaghi A. Silani G. (2016). Emotional egocentricity bias across the life-span. *Front. Aging Neurosci.* 8:74. 10.3389/fnagi.2016.00074 27199731 PMC4844617

[B88] Robinson J. A. Taylor L. R. (1998). “Autobiographical memory and self-narratives: a tale of two stories,” in *Autobiographical Memory*, eds Thompson C. P. Herrmann D. J. Bruce D. Read J. D. Payne D. G. Toglia M. P. (Mahwah, NJ: Erlbaum), 125–143.

[B89] Seger C. A. Stone M. Keenan J. P. (2004). Cortical activations during judgments about the self and an other person. *Neuropsychologia* 42 1168–1177. 10.1016/j.neuropsychologia.2004.02.003 15178169

[B90] Sestir M. Green M. C. (2010). You are who you watch: identification and transportation effects on temporary self-concept. *Soc. Influ.* 5 272–288. 10.1080/15534510.2010.490672

[B91] Silani G. Lamm C. Ruff C. C. Singer T. (2013). Right supramarginal gyrus is crucial to overcome emotional egocentricity bias in social judgments. *J. Neurosci.* 33 15466–15476. 10.1523/jneurosci.1488-13.2013 24068815 PMC6618458

[B92] Simony E. Honey C. J. Chen J. Lositsky O. Yeshurun Y. Wiesel A. (2016). Dynamic reconfiguration of the default mode network during narrative comprehension. *Nat. Commun.* 7:12141. 10.1038/ncomms12141 PMC4960303 27424918

[B93] Singer J. A. Blagov P. (2004). “The integrative function of narrative processing: autobiographical memory, self-defining memories, and the life story of identity,” in *Studies in Self and Identity. The Self and Memory*, eds Beike D. R. Lampinen J. M. Behrend D. A. (New York, NY: Psychology Press), 117–138.

[B94] Singer J. A. Bluck S. (2001). New perspectives on autobiographical memory: the integration of narrative processing and autobiographical reasoning. *Rev. Gen. Psychol.* 5 91–99. 10.1037/1089-2680.5.2.91

[B95] Slater M. D. Johnson B. K. Cohen J. Comello M. L. G. Ewoldsen D. R. (2014). Temporarily expanding the boundaries of the self: motivations for entering the story world and implications for narrative effects. *J. Commun.* 64 439–455. 10.1111/jcom.12100

[B96] Slater M. D. Rouner D. (2002). Entertainment—education and elaboration likelihood: understanding the processing of narrative persuasion. *Commun. Theory* 12 173–191. 10.1093/ct/12.2.173

[B97] Steinbeis N. Singer T. (2014). Projecting my envy onto you: neurocognitive mechanisms of an offline emotional egocentricity bias. *NeuroImage*, 102, 370–380. 10.1016/j.neuroimage.2014.08.007 25117603

[B98] Steinemann S. T. Iten G. H. Opwis K. Forde S. F. Frasseck L. Mekler E. D. (2017). Interactive narratives affecting social change. *J. Media Psychol.* 29 54–66. 10.1027/1864-1105/a000211

[B99] Stolte J. F. Fender S. (2007). Framing social values: an experimental study of culture and cognition. *Soc. Psychol. Q.* 70 59–69. 10.1177/019027250707000107

[B100] Summerfield J. J. Hassabis D. Maguire E. A. (2009). Cortical midline involvement in autobiographical memory. *Neuroimage* 44 1188–1200. 10.1016/j.neuroimage.2008.09.033 18973817 PMC2625448

[B101] Swanson R. W. Gordon A. S. Khooshabeh P. Sagae K. Huskey R. Mangus M. (2017). An empirical analysis of subjectivity and narrative levels in Weblog storytelling across cultures. *Dialogue Discourse* 8 105–128. 10.5087/dad.2017.205

[B102] Szameitat A. J. Shen S. Sterr A. (2007). Motor imagery of complex everyday movements. An fMRI study. *Neuroimage* 34 702–713. 10.1016/j.neuroimage.2006.09.033 17112742

[B103] Tabei K.-I. (2015). Inferior frontal gyrus activation underlies the perception of emotions, while precuneus activation underlies the feeling of emotions during music listening. *Behav. Neurol.* 2015 529043. 10.1155/2015/529043 PMC4609410 26504353

[B104] Uddin L. Q. Kinnison J. Pessoa L. Anderson M. L. (2014). Beyond the tripartite cognition-emotion-interoception model of the human insular cortex. *J. Cogn. Neurosci.* 26 16–27. 10.1162/jocn_a_00462 23937691 PMC4074004

[B105] Uddin L. Q. Nomi J. S. Hébert-Seropian B. Ghaziri J. Boucher O. (2017). Structure and function of the human insula. *J. Clin. Neurophysiol.* 34 300–306. 10.1097/wnp.0000000000000377 28644199 PMC6032992

[B106] van der Heiden L. Scherpiet S. Konicar L. Birbaumer N. Veit R. (2013). Inter-individual differences in successful perspective taking during pain perception mediates emotional responsiveness in self and others: an fMRI study. *Neuroimage* 65 387–394. 10.1016/j.neuroimage.2012.10.003 23063451

[B107] Van Laer T. De Ruyter K. Visconti L. M. Wetzels M. (2014). The extended transportation-imagery model: a meta-analysis of the antecedents and consequences of consumers’ narrative transportation. *J. Consum. Res.* 40 797–817. 10.1086/673383

[B108] Vogeley K. Fink G. R. (2003). Neural correlates of the first-person-perspective. *Trends Cogn. Sci.* 7 38–42. 10.1016/s1364-661300003-7 12517357

[B109] Watanabe T. Yagishita S. Kikyo H. (2008). Memory of music: roles of right hippocampus and left inferior frontal gyrus. *Neuroimage* 39 483–491. 10.1016/j.neuroimage.2007.08.024 17905600

[B110] Weiss J. K. (2015). *Maybe Medium Does Matter: Considering Differences in Individual’s Trait Empathy and the Effect on Narrative Transportation.* Buffalo, NY: State University of New York.

[B111] Yao S. Becker B. Geng Y. Zhao Z. Xu X. Zhao W. (2016). Voluntary control of anterior insula and its functional connections is feedback-independent and increases pain empathy. *Neuroimage* 130 230–240. 10.1016/j.neuroimage.2016.02.035 26899786

[B112] Yeshurun Y. Swanson S. Simony E. Chen J. Lazaridi C. Honey C. J. (2017). Same story, different story: the neural representation of interpretive frameworks. *Psychol. Sci.* 28 307–319. 10.1177/0956797616682029 28099068 PMC5348256

[B113] Zaki J. Davis J. I. Ochsner K. N. (2012). Overlapping activity in anterior insula during interoception and emotional experience. *Neuroimage* 62 493–499. 10.1016/j.neuroimage.2012.05.012 22587900 PMC6558972

[B114] Zhang S. Chiang-shan R. L. (2012). Functional connectivity mapping of the human precuneus by resting state fMRI. *Neuroimage* 59 3548–3562. 10.1016/j.neuroimage.2011.11.023 22116037 PMC3288461

